# Reach the Person behind the Dementia - Physical Therapists' Reflections and Strategies when Composing Physical Training

**DOI:** 10.1371/journal.pone.0166686

**Published:** 2016-12-01

**Authors:** Anncristine Fjellman-Wiklund, Ellinor Nordin, Dawn A. Skelton, Lillemor Lundin-Olsson

**Affiliations:** 1 Department of Community Health and Rehabilitation, Physiotherapy, Umeå University, Umeå, Sweden; 2 School of Health and Life Sciences, Institute of Applied Health Research, Glasgow Caledonian University, Glasgow, United Kingdom; Nathan S Kline Institute, UNITED STATES

## Abstract

Dementia is a disease characterized by cognitive impairment and physical decline that worsens over time. Exercise is one lifestyle factor that has been identified as a potential means of reducing or delaying progression of the symptoms of dementia, maximizing function and independence. The purpose of this study was to explore physical therapists’ (PTs) experiences and reflections on facilitating high-intensity functional exercise with older people living with dementia, in residential care home settings. The study used a qualitative design based on interviews, individually or in small groups, with seven PTs engaged as leaders in the training of older people with dementia. The interviews were analyzed with a modified Grounded Theory method with focus on constant comparisons. To increase trustworthiness the study used triangulation within investigators and member checking. The core category *“Discover and act in the moment—learn over time”* reflects how the PTs continuously developed their own learning in an iterative process. They built on previous knowledge to communicate with residents and staff and to tailor the high intensity training in relation to each individual at that time point. The category *"Be on your toes"* highlights how the PTs searched for sufficient information about each individual, before and during training, by eliciting the person's current status from staff and by interpreting the person's body language. The category *"Build a bond with a palette of strategies"* describes the importance of confirmation to build up trust and the use of group members and the room to create an interplay between exercise and social interaction. These findings highlight the continuous iterative process of building on existing knowledge, sharing and reflecting, being alert to any alterations needed for individuals that day, communication skills (both with residents and staff) and building a relationship and trust with residents in the effective delivery of high intensity functional exercise to older people living with dementia in care settings.

## Introduction

Dementia is a degenerative disease with no known cure, and symptoms get worse over time such as cognitive impairment, difficulty communicating and functional declines. However, people living with dementia can still have a good quality of life throughout the dementia journey, provided the right long-term care plan is in place and being delivered [1]. Half of all older people who need personal care have dementia, and 8 out of 10 older people in residential care facilities are living with dementia [1]. The worldwide cost of dementia care is currently over US $600 billion, or around 1% of global GDP [1]. Exercise is one lifestyle factor that has been identified as a potential means of reducing or delaying progression of the symptoms of dementia, maximizing function and independence [2, 3]. A Cochrane Review found promising evidence that exercise programs can significantly improve cognitive functioning of people with dementia and their ability to perform daily activities, but no significant effect on mood, including challenging behaviors and depression [4].

Despite the evidence that exercise is beneficial for older people with dementia, and a variety of guidelines and recommendations advise health and public health professionals to engage and refer people living with dementia to activity programs, there is still a dearth of evidence on how best to work and engage with people in care settings [5–9]. To date, no studies have considered those working in delivery of exercise or physical rehabilitation activity. There has, however, been research published on professionals’ views of effective ways to communicate and engage with people with dementia within interventions that focus on movement and activity. One explored geriatric nurse assistants’ views and experiences of the motivators and barriers to engaging people living with dementia in functional activities and exercise before undertaking a pilot RCT [10]. However, the nurse assistants were not charged with delivering exercise, instead they were trying to get the residents moving more often. The other study describes the experiences of the healthcare professionals in delivering a multicomponent psychosocial program for people with dementia and their families in Norway [11]. This intervention did not include an exercise component, and was aimed at those still living in the community, but did describe the challenges, new knowledge and service development from the perspectives of the intervention providers.

To support the effective implementation of exercise and rehabilitation for people living with dementia it is important to understand how leaders of exercise have learnt from their experience and what they think best supports effective exercise delivery. In order for recommendations on increasing activity in people living with dementia to lead to implementation of programs, leaders of exercise need to understand the challenges, barriers and opportunities for effective behaviour change in this specific population group. HIFE, High Intensity Functional Exercise program, is an effective exercise intervention delivered by physical therapists (PTs) with older people living with dementia in care home facilities [12, 13].

The purpose of this study was therefore to explore PTs’ experiences and reflections on providing and facilitating high-intensive functional exercise with older people living with dementia living in residential care facilities. The results of the interviews with PTs will allow future leaders of exercise with people living with dementia to capitalize on prior knowledge and strategies that were effective in this population living within a care setting, in order to facilitate effective exercise for health and functional benefits.

## Methods

### Research design

This study had a qualitative design, based on interviews with PTs engaged as leaders in an exercise intervention for older people with dementia living in residential care facilities. The intervention itself has previously been evaluated in a cluster-randomized controlled trial (cRCT), the UMDEX study [13]. The interviews were analyzed with a modified Grounded Theory (GT) method with focus on constant comparisons [14] since GT is considered as especially useful when illustrating a process over a longer period, such as exercise interventions like UMDEX. GT is also suitable when studying complex factors that influence health and illness when little knowledge is at hand in a field [14, 15], which is the case when PTs deliver exercise programs directed to people with dementia.

### Ethical statement

This study is part of the UMDEX study, the cluster-randomized controlled trial (cRCT) with the primary aim to evaluate effects of high-intensity functional exercise on independence in activities of daily living (ADLs) in older people with dementia [13]. The study protocol for the cRCT (ISRCTN31767087) is published on the ISRCTN registry website (http://www.isrctn.com). The study was approved by the Regional Ethics Vetting Board in Umeå, Sweden (2011-205-31M).) This interview study only included the PTs who acted as exercise leaders. We did not seek ethical approval for this qualitative part of the study but all PTs gave oral and written informed consent. No sensitive health-related data was obtained from the PTs and confidentiality and anonymity was maintained for the people with dementia and staff they spoke about, and the benefits were estimated to be greater than the risks. Possible benefits were a deeper reflection and learning during the interviews about how to act as an exercise leader for groups of people with dementia. There was a risk that the participant's integrity would be violated during the interview. To minimize this risk, the interviews were conducted in small groups by a suitable trained and experienced interviewer and the interview guide was based on open-ended questions. Thus, the PTs could select themselves and what they wanted to tell in that situation. There was no coercion to be involved and they were also invited to come back if they wanted to. The PTs were asked if they were willing to be acknowledged in the paper and they all approved.

### The context

The exercise intervention was based on the High-Intensity Functional Exercise (HIFE) program [12]. The exercises, administered in small groups but tailored to each person with dementia, were performed in functional weight-bearing positions, similar to everyday situations such as reaching an object in standing and rising up from a chair. The intensity of the balance and strength exercises was intended to be high, and progressed through increased load and difficulty. The PTs chose exercises throughout the intervention period to meet levels of functional status, cognition, behavioral and psychological symptoms of dementia, as well as changes in health status. Pairs of PTs were responsible for six different exercise groups; each exercise group met five times per fortnight. After each training session the PTs completed a structured protocol for each person with dementia documenting attendance, selection of exercise tasks, achieved intensity, motivation, adverse events (e.g. occurrence of symptoms such as pain or discomfort brought on or worsened during the activity session). This summary gave the pair of PTs an opportunity to discuss successes, difficulties and any amendments for the next session.

The physical exercise took place within the resident′s facility but outside the care unit. Before each group session, furniture and equipment were arranged by the PTs. The 93 residents in the exercise arm of the study, 70 women and 23 men with a mean age ± standard deviation 84.4±6.2 years, lived in 16 residential care facilities in northern Sweden. These facilities included general as well as dementia units, with private apartments or private rooms with staff at hand and access to on-site nursing. All had a dementia diagnosis with moderate to severe cognitive impairment (Mini-Mental State Examination [16] score: mean 15.4±3.4). They had a slow gait speed (0.47±0.21 meters/second with walking aid if needed) and there was a wide range of dependency in personal activities of daily living (dressing, grooming, transfer etc); all were dependent in at least two activities, while some were dependent in almost all activities (Barthel Index [17]: mean 10.7±4.5). The staff at the care unit were initially asked to bring the person to the exercise venue, but to facilitate attendance it became more common for the PTs to get the people to the group themselves. As the PTs only met the person during a cross section of the day, 2–3 times a week, they were dependent on the staff at the care unit to provide them with an update regarding any health-related issues for individuals that might interfere with ability to exercise since their last visit.

The PTs responsible for delivering the physical exercise undertook also the assessments in the cRCT. The blinding to group activity was attained by the PTs working pair-wise and being assigned to certain residential facilities for the intervention and other facilities for the assessments. Thus, from the start of the assessments to the completion of a follow-up 3 months after the intervention had ended, no information that could reveal allocation of a person was to be shared outside the pairs of PTs.

### Participants and data collection

Seven PTs worked with the UMDEX intervention, four worked full time and three worked part time, corresponding to six full time positions. During spring 2013, before ending the intervention, all seven PTs, were asked to participate in qualitative interviews, in smaller groups or individually, to allow for continued blinding to the intervention groups. Four women and three men, 26–54 years of age gave their informed consent and participated in this qualitative study. They had a wide range of years since qualification as PTs (range of 3 to 20 years), with a range of 0.5 to 13 years clinically in the field of geriatrics. Some had PhDs in the area of rehabilitation in geriatrics and some had experience of living or working with people with dementia, alongside their clinical experience.

In total, there were five interviews with the seven PTs. They all worked in the same intervention but one PT worked in two pairs. One group interview with three PTs, together with two interviews with pairs of PTs were done. Since the PTs in the group interview had so much to tell, an additional individual interview together with an interview with two PTs were made. The group/pair interviews were inspired by focus-group principles [18], collecting data from people’s own experiences and views as an established way of collecting qualitative data. Focus groups can help participants to explore and clarify their specific topics and each participant may highlight their own attitude and priority during the discussion [19, 20]. Focus groups construct the participants as active collaborators in order to generate new knowledge, allowing the members of the group to interact and influence each other.

**Fig 1:**
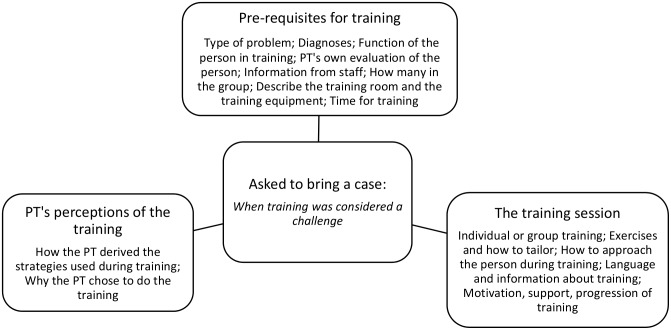
Topics in the semi-structured interview guide used with physical therapists training older people with dementia.

Prior to the interviews the PTs were asked to bring a case study, where the training was considered a challenge, to start the discussion. To facilitate the interviews an interview guide with semi-structured questions in three themes: ‘Pre-requisites for training’, ‘The PT’s perceptions of the training’, ‘The training session’ was used (Fig 1). Between the interviews minor adjustments were made to the questions, so that information provided in one interview could be taken into consideration in a following. Each group interview was led by a moderator (AFW), with support from an observer (LLO or PP), who aimed at creating an atmosphere of confidence and enabled participants to talk freely. The moderator started by asking each participant to talk about their case study. The observer took notes throughout and at the end of the interview summarized the group discussion. The individual interview was moderated by AFW only. All interviews were performed in a conference room at the university. The group interviews lasted between 40–75 minutes, the individual interview 35 minutes, and were audio-recorded and transcribed verbatim, in Swedish. All identifiable information was first removed before translation into English. One author (DS) native speaker in English, translated the manuscripts into English using Google translate and multiple discussions within the author team meetings confirmed the exact meaning and wording as many phrases were ambiguous. Constant reflection back to the original Swedish manuscripts by the authors ensured that the content was not changed due to the translation. Discussion within the whole authorship confirmed the translations of the content of the quotes and the final quotes used.

### Data analysis

The interviews were analyzed using a modified Grounded Theory (GT) method with a focus on constant comparisons [14, 15]. The transcripts were carefully and repeatedly read in parts and as a whole during the analysis process. Ideas that came up relating to the emerging results were continuously written down in memos and used in the analysis. The analysis started with open coding by the first author (AFW), meaning that the transcripts were read line-by-line and paragraph by paragraph and important information was given codes relating to the text. All authors contributed to the next step, codes with similar content were compiled to construct sub-categories and categories on a more abstract level. The categories were then compared with how they related to each other in an axial coding. During the analysis of the categories and the connections between them, a core category, which was central to all data, was identified. During all steps of the analysis each author, performed a mutual comparison and a final negotiated outcome between all authors was achieved. In the last step, a model was constructed to illustrate the whole process.

Three of the authors are physical therapists (AFW, EN, LLO) and one author is an exercise physiologist (DS). All have experience in qualitative research. In order to increase trustworthiness, the result was discussed repeatedly in the research group and eventually presented and discussed with all PTs in a member check [21]. The PTs recognized themselves and their narratives in the description of the categories and the content of the core category but suggested some small changes of the wording of the core category, which were implemented.

## Results

**Fig 2:**
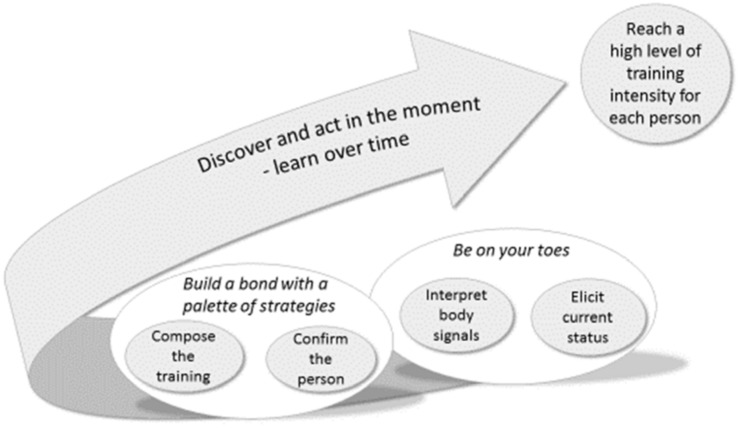
Model of physical therapists’ reflections and strategies when facilitating high-intensity functional exercise with older people living with dementia, in residential care home settings. Results emerging from the Grounded Theory analysis.

The core category ***Discover and act in the moment—learn over time***, together with two categories, ***Be on your toes*** and ***Build a bond with a palette of strategies*** explore the PTs’ experiences and reflections during the process of providing and facilitating high-intensity functional exercise with older people with dementia, living in care home settings. (Table 1) (Fig 2).

**Table 1:** Illustration of the analysis process from codes to core category.

Examples of codes	Sub-category	Category	Core category
Uncertainty creates responsiveness	• Elicit current status	• Be on your toes	• Discover and act in the moment—learn over time
Accuracy in questions			
Specific questions for better answers			
One “read” if they didn’t “tell”	• Interpret body signals		
Gestures			
Light in their eyes			
Dementia hides symptoms			
Check pulse			
Health observer			
Get it to work all the time	• Confirm the person	• Build a bond with a palette of strategies	
Find new ways			
When to stop the training?			
Eye contact			
Body contact			
See possibilities			
Group advantages	• Compose the training		
Positive group energy			
Cohesive group			
Not too large a group			
Interactions between participants			
Equipment in the right place			
No rush, calm down			
Motivate			
Repeat			
Convince			
Adapt			
Talk about other things			

### Discover and act in the moment—learn over time

The core category ***Discover and act in the moment—learn over time*** describes how the PTs continuously developed their own learning process. They built on communication skills with people with dementia and staff and also to tailor training at a high-intensity level for each individual.

The PTs showed great respect, their intent was that each individual should have fun and feel good about the training, whilst at the same time the training should be at a high-intensity level. The PTs felt enthusiastic about providing training for people with dementia. The training as such was a *“trial and error situation”* and a challenge. The PTs were *“curious to try new ways”* to accomplish training for people with dementia. They saw themselves as *“detectives”*, *“problem solvers”* and *“advocates”* for these people. They were driven by the overall goal of *“doing good and doing right”*, with respect, for people with dementia as they too had *“the right to move”*, *“to exercise”* and *“the right to rehabilitation”*, since they *“seldom get anything”*. They searched for optimal actions and creative solutions and took note of the small details in the training. They continually learned from their experience, which varied from session to session, and integrated the new knowledge to their existing knowledge in an iterative process.

I always try to solve problems directly. And think of the possibilities…to find possibilities to reach through to the persons…finding new ways, all the time. And I can accept if it fails…I did the best I could. It depends on me, not the persons in the training…it’s never their fault

(Interview 4)

The category ***Be on your toes*** highlights how the PTs, in order to develop training, searched for information about each individual, before and during training, by checking the person's current health status and by interpreting the person's body language. The category ***Build a bond with a palette of strategies*** describes how the PTs made efforts to build up trust through confirmation between themselves and the residents and through the placement of group members within the room to create an interplay between exercise and social interaction.

### Be on your toes

The category ***Be on your toes*** was constructed by the sub-categories ***Elicit current status*** and ***Interpret body signals***, which illuminate when the PTs searched for information about each person, before and during the training sessions.

#### Elicit current status

In order to prepare for and obtain optimal conditions for each training session, the PTs felt it important to be aware of each person’s physical and mental health status at all times. This was facilitated by talking both to the person her/himself and to the staff at the facility in an *“interacting way”*. The PTs asked *“directed specific questions about specific individuals to specific staff”*. Dementia was experienced as *“hiding”* other diseases and symptoms, therefore the questions needed to be specific about each person. The questions dealt with medical and emotional events or situations that might interfere with the person’s ability to focus and be active across the group session, such as sleep, pain, anxiety, medication or general health. The PTs way of asking these questions evolved in a process over time and became gradually learnt, specific and selected. The way of asking was built on their general competence of dementia and their own experiences of working with people with dementia and the peer discussion between the pairs of PTs. Asking general questions to staff such as”How does he/she feel today?” did not give enough information to prepare for the high intensity training. Examples of specific questions were instead: *“Did he sleep well*?*”*, *“Has she been confused*? *“Has she had angina during …*?*” Has he had pain today*? The learning process of asking and gaining important insights was extensive and they had the *“courage to try”* and perceived it as *“a challenge”*. If the PTs could not get the information they needed they *“felt frustrated”*.

If you ask the staff [about the person] and they say: “It’s ok” you’re not satisfied with that. You start asking more specific…”Has she had more knee pain? Has she been up today?”

(Interview 4)

They [the persons] express themselves in a different way .to look worried means something else than you may think…and you have to [ask] “Do you have pain?” and often they do.…to be more open, to listen to what they can express without using words

(Interview 1)

#### Interpret body signals

The PTs also prepared and planned for each training session through their own contact and interpretation of each person. Their goal for the interpretation was to prepare for training at a high level at each training session and *“not go over the border”* (acceptable limit) for each person. The interpretation was based on being *“here and now”* and they *“tuned in themselves”*. The PTs paid closed attention to each person’s verbal and non-verbal communication. They looked at gestures, facial expressions, body posture, if the person was sitting very still or moving around, if they seemed happy or sad and how the mood eventually shifted during the training. The PTs tried to interpret if the people seemed interested in and interacted with other people in the training room. They were attentive to anyone expressing fatigue or if the load or intensity became too high. The PTs noticed the gaze, *“how the person looks back at you”* or if the person *“drifts away”*. The PTs had a *“strong belief”* and relied on their own interpretations.

…to have the time to read face expressions, and look at movement quality and see. How challenging is this exercise? Is it a good exercise and how is it performed?

(Interview 3)

It is in the gaze, and maybe gestures, that they are with you. They can sit very still and look into nowhere. but at the end of the session they are here and now and look at you and are interested in others and comment

(Interview 1)

### Build a bond with a palette of strategies

The category ***Build a bond with a palette of strategies*** was constructed by two sub-categories; ***Confirm the person*** and ***Compose the training***. It describes how trust was built between the person with dementia and the PT and explores how the PTs set up the group training.

#### Confirm the person

During all training sessions the PTs were aiming at”*reaching the person behind the dementia”* through *“creating a relationship”* with the person so the person felt *“seen*, *met and confirmed”* and *“boosted as a person”*. The PTs wanted to *“find the hook”* for each person, meaning they tried to find an individual and personal topic to talk to each person about, such as recollections of past events and old memories that were meaningful to the individual. The PTs wished to *“create trust”* and thereby confidence in the personal meeting and a feeling for the person of being *“safe in the training situation”*. Every session was aimed to be an *“optimal situation”* with *“positive feedback during exercise”* and *“talk before and after the exercise”*. After the training session they wanted each person to feel *“empowered”*, *“proud of what they have accomplished”*, that they *“had succeeded despite the dementia”* and had *“had a fun moment”*.

You’re looking for things, to get in contact [with the person] to make it work all the time, in some way

(Interview 4)

#### Compose the training

The PTs *“used the group members”* and *“arranged”* the training with certain strategies to make the persons feel confident to perform on a high level and still have fun. Each training session was built as an *“interplay between physical exercise and social interaction”* in a *“calm and stress-free”* environment. During the training sessions the persons were to *“recognize the physical therapist (person*, *voice)”*,*”recall the exercise”* and the *“feeling of the exercise and training session”*.

The PTs described the optimal training group as having four to six people and two PTs, in order to avoid feelings of stress, for the person training and the PTs themselves. The training room would ideally be spacious to also accommodate exercise equipment. Depending on each person’s status for the day, they were placed in certain positions in the room. The PTs placed people in a way they thought was comfortable for every individual. Friends could sit beside each other and some who liked to cheer and push others could be placed close to someone who needed encouragement. Some could be turned facing the wall for better concentration while they performed an exercise task.

If you have four persons in a group and two leaders…you do have time. They have time to exercise and you can chat and you can ‘meet them’. If you have more people it’s more stressful, you don’t get the good meeting and you can’t train everything you would like to do

(Interview 2)

The good training rooms we have had have been spacious. Where you can turn some away from the group, without making them feel too close to the wall… and (the training room) has windows. And possibilities to walk, not in a long corridor where people walk around, since our participants may have problems with concentration and they get distracted by something…a place where you can place some boxes (as training equipment)

(Interview 3)

The PTs’ judgment of the intensity of each exercise at every training session had to be achievable for each person and the PTs’ wanted to facilitate and stimulate the residents towards the goal; to have a high intensity balance and strength training, progressing over time. They used *“all senses- seeing*, *hearing*, *speaking*, *touching”* in the training, to come *“closer to each person’s boundary”* (limit) of what was possible in the training. The exercise instructions had to be *“simple and tailored to each person”*. The PTs often repeated the instructions and motivated and inspired the persons to perform to a high level and intensity. They pushed and *“pep-talked”* some people to *“keep going”* with the training, whilst some they needed to hold back to avoid over exertion. The PTs kept close trying to have *“eye contact with each person”*. They also supported and reinforced the exercises with positive verbal feedback and with physical contact for those who wanted that. Before they touched the person they were careful to let the person “*invite*” the physical contact (touch). The PTs were very concerned about showing respect for each person, whilst foreseeing potential problems such as people disturbing each other, getting angry or being sad.

You want to see the individual and you want to progress the exercise the whole time and not just routinely put on weight belts or choose exercise…you need some time to think and reflect

(Interview 3)

When it comes to (physical) contact with the elderly, I’ve found out that I like to lay a hand on the shoulder when you talk to them, and not all elderly like it. Your really have to hold back, because… the woman I told you about … from the beginning I made a catastrophic mistake and put my hand on her shoulder and she said: "Do not touch me!". I don’t know what triggered it. I kind of learned that I don’t touch her until she has touched me. Just such a thing has made it work better.

(Interview 5)

## Discussion

This study is the first to explore the views and experiences of PTs delivering an exercise program to older people living with dementia in care settings. It provides useful insights into successful strategies and how future exercise leaders working with this vulnerable population might best achieve effective adherence and outcomes to a high intensity exercise program with proven benefits.

The core category *Discover and act in the moment—learn over time* shows that the PTs gradually learned from experience and they integrated this new knowledge with their existing knowledge in an iterative process. They worked in pairs, and after each training session they completed a protocol for each person with dementia including exercise tasks, achieved intensity, motivation, and adverse events (symptoms or discomfort). This was a moment that facilitated reflection based on questions like “What happened?” “Why did it happen?”. They also had the opportunity to share their thoughts with a colleague directly after each exercise session. A similar process took place when the PTs learned how to put questions to staff to elicit the person’s current status.

Before the planned group meetings, with all seven PTs, they further reflected on their experiences because they had to carefully plan what to bring to the discussion and how to express themselves in order to describe situations at a more abstract level in order to maintain blinding within the study objectives. These shared reflections enabled the experience to be integrated into their “bank of knowledge” in a wider sense than relying only on self-reflection. These processes can be understood through Schön’s model [22] concerning *reflection-on-action* and *reflection-in-action*. Reflection-on-action means learning from unique situations in practice by thinking through the issues, trying new ways, restructuring actions, altering practice and sharing with colleagues. This process was described by the PTs as being ‘*a detective curious to try new ways*, *guided by trial and error*’. The PTs gave examples of reflection-in-action when they changed their way of acting as a response to minute-by-minute changes in an ongoing situation, dependent on how they interpreted the s verbal and non-verbal communication.

In the category *“Be on your toes”* the PTs describe that they felt dependent on the staff at the care homes to give them an update, from their last visit, about the persons’ health-related issues that could interfere with the ability to exercise. This was because the PTs visited the person’s s’ facilities two or three times per week only and the dementia disease involves both difficulty to verbally describe situations and to remember what has happened. Comorbidity is common among people with dementia in care homes; it is estimated that the majority (96.8%) have at least two comorbidities in addition to their dementia [23]. A strong association between medical comorbidity and cognitive status in Alzheimer’s disease, cause of 60–70% of cases of dementia, has been shown, meaning that a lower cognitive status is more likely to imply more comorbidity [24]. Furthermore, those living with dementia often have a fluctuating status, including exacerbations of chronic conditions and new physical problems. For example, one study showed that almost all (94%) nursing-home residents with dementia over a period of six weeks had changes to their health condition status, and many of the symptoms were considered treatable or reversible [25]. Early recognition and treatment of new conditions and exacerbations of ongoing conditions is thus of great importance [26]. It has been emphasized by assistant nurses that teamwork is a key component to engage people with dementia in activities, partly because of the opportunities to report and discuss their changing status [10]. The PTs were aware that they had to *interpret body signals* like facial expressions, speech intonation, and body language and that they only met the person with dementia during a short time period in the week. They developed skills to *elicit current status* from staff. Staff that work close to the people with dementia have a very important role—that they not always are aware of—in the implementation of exercise programs. They can pay attention to emerging symptoms and interpret changes around the clock in activity patterns, interpersonal interactions, and mental status in addition to facial expressions, speech intonation, and body language.

The PTs were keen to *build a bond with a palette of strategies* and they created a relationship with each person with dementia so that he/she should feel welcomed and safe during the training session. The nature of the cognitive and functional impairments can make it difficult for people with dementia to satisfy their own needs and this can impact on well-being [27, 28]. Indeed, it has been found that increasing interactions that address residents’ identity has a positive impact on their well-being [28]. Memory loss is a threat to identity for people with dementia, but to be reminded by others about important and meaningful situations in the past can help maintain identity. The PTs in this study *confirmed the person*, when they made efforts to “*reach the person behind the dementia*” and included small talk in the exercise program about topics that conceived to be meaningful for each person with dementia, often about memories from the past. The importance of using each person’s past to motivate people with dementia to be actively involved in daily activities has also been emphasized by assistant nurses in care homes [10]. Overall, the PTs strived in different ways to meet psychological needs so that the people with dementia could be proud of what they had accomplished. They talked of their willingness to make the session fun and this has been confirmed by other health professionals working on psychosocial interventions with people living in the community with their carers [12]. The importance to pay close attention to the their mood is further reinforced by findings that people with Alzheimer Disease experience prolonged states of emotion that persist long after events that they have forgotten [29]. The PTs felt that the sessions would be most effective if held with smaller numbers of people with dementia and two PTs, so that they could have more time to tailor and personalize the program for each individual and having two leaders was also recognized as important by health care professionals working with a psychosocial intervention [12]. Perhaps this also reinforces the shared learning that delivering interventions in pairs can provide for the professionals as well as the more interactive and diverse experience for those within the interventions.

In order to enable each person with dementia to succeed in their challenging exercise tasks the PTs in this study *composed the training* for each person based on their cognitive and physical ability, as well as on the placement of the other members of the group and in the physical room, including removing unwanted stimuli. This is in accordance with the system-theoretical approach to motor control which includes an interaction between the person-environment-task, as suggested by Woollacott & Shumway-Cook [30]. The HIFE program consists of exercise tasks which are functional and based on everyday-tasks, for example stand up and sit down in a chair, or step up and down on a stair step [13]. The PTs did not express their thoughts in the interviews about the selection of the HIFE exercise tasks for particular persons, instead focused on how they implemented the HIFE-program in this group, taking the cognitive and perceptual impairments into account. The PTs used communication skills and motivational techniques tailored to each person with dementia including short and clear verbal cues to guide activity, as well as bodily movements such as gestures, gestures, eye contact and touch, with the aim to positively reinforce their actions. The importance of these specific skills are not unique to PTs but are, of course, important for all staff and professionals in all settings caring for people with dementia. Indeed, a review of communication skills training in dementia care showed significant improvements in the quality of life and wellbeing of people with dementia following increased positive and person-centred interactions in various care settings [31].

### Strength and weakness of the study

A strength of this study was the richness of the interview data. All PTs providing and facilitating HIFE training within the UMDEX trial participated in the interviews. The PTs represented a wide range of experiences in the field of geriatrics and in training of people with dementia, some with long and extensive experience as PTs working with older people, some with less experience. We consider this as an advantage, as it allowed different kinds of experiences to be discussed. There were no obvious signs of imbalance in opportunities for the PTs with less experience to give their points of view during the interviews.

Another strength of the study was that the authors represented different professional perspectives and languages, including both an “insider” and an “outsider perspective”. The first author (AFW) had no experience of working in geriatrics and training with people living with dementia and thus could be seen as an outsider in the field of geriatrics. She has extensive experience of qualitative methods and was the one who did the first coding and interpretation of the data material. Two authors represented an “insider perspective”; LLO was part of the steering group of UMDEX and LLO and EN participated as assessors in UMDEX. DS represents both an “insider and outsider perspective”, with extensive experiences from the field of geriatrics and from training frailer older people with and without dementia, but was not part of the UMDEX study. All authors performed independent analyses, which were thoroughly discussed and refined until consensus was achieved, being aware of the potential risk for bias inherent in our roles and languages as “insiders and outsiders”, we made effort to monitor our interpretations. The result was also presented and discussed with the PTs, to help guard against over-interpretation of the results and improve trustworthiness [21].

The low number of participants in each group interview could be considered a limitation for reaching saturation. A focus group is recommended to consist of four to eight participants [20]. Due to the avoidance of un-blinding, this was not possible in this study. The intention was to look upon each pair of PTs in the UMDEX as a unit and thus let them discuss their experiences together. One PT worked in two units and therefore an individual interview was required to avoid un-blinding. However, all PTs involved in the UMDEX intervention were included in this study, they were all experts, in different fields of geriatrics and the interviews gave rich data.

### Implications for practice

It is important that managers, staff and others are aware of that people living with dementia in care home settings are able to carry out high-intensity functional exercise training when they are supervised by skilled and knowledgeable exercise supervisors. Supervised training is necessary for people with dementia living in care settings, not just because of the risk of adverse events with fluctuating health and problems with communication, but also to provide optimal training with their health and abilities that day. PTs have to look behind the dementia disease, both regarding the person’s previous experience and comorbidities, and to have communication skills alongside skills in clinical reasoning to meet the needs of the person and to empower the person to achieve and feel good about the training. No exercise program fits all and each session must be tailored for each person. Exercise leaders working with people with dementia, in care settings, have to alter this tailoring from session to session and this requires skill and expertise to make ‘on the spot’ decisions to ensure both the safety and the effectiveness of training. They also need to be able to ‘see beyond the dementia’ and be able to interpret body language before and throughout sessions. Care staff knowledge and interest is important even though they do not take an active part in the exercise program. Their experience and information about current health status of people with dementia allows the PTs to build “today’s platform for decision-making” so that the PTs can find the optimal intensity level for exercise training residents with dementia.

## Conclusion

It is a complex task to be an exercise leader of people with dementia in care settings. To guide effective training knowledge about how to select and to perform exercise tasks and about the dementia disease are vital. The ability to be flexible in approach, tailor and personalize communication (verbal and non-verbal), optimize the use of the group and the room, and to build a successful collaboration with the staff is vital to success. The process of knowledge gains through reflection- on-action and in-action were important to the PTs in delivering optimal and progressive training to residents in care settings living with dementia.
